# Combined Use of Indirect ELISA and Western Blotting with Recombinant Hepatocellular Carcinoma-Associated Antigen 59 Is a Potential Immunodiagnostic Tool for the Detection of Prepatent *Haemonchus contortus* Infection in Goat

**DOI:** 10.3390/ani9080548

**Published:** 2019-08-13

**Authors:** Muhammad Ali-ul-Husnain Naqvi, Sana Zahra Naqvi, Muhammad Ali Memon, Kalibixiati Aimulajiang, Muhammad Haseeb, Lixin Xu, Xiaokai Song, Xiangrui Li, Ruofeng Yan

**Affiliations:** MOE Joint International Research Laboratory of Animal Health and Food Safety, College of Veterinary Medicine, Nanjing Agricultural University, Nanjing 210095, Jiangsu, China

**Keywords:** *Haemonchus contortus*, rHc-HCA59, prepatent diagnosis, indirect ELISA, western blotting

## Abstract

**Simple Summary:**

The accurate and early diagnosis of *Haemonchus contortus* infection is crucial for effective control. Early stage detection of *H. contortus* infection has not been attempted in goat, even though both immature worm and fourth larval stage are blood sucking. This study was carried out to detect the *H. contortus* infection during early stage in goat. The results of this study assessed that rHc-HCA59 could detect the antibody in *H. contortus* infected goats’ sera during early period with good sensitivity and specificity using immunodiagnostic techniques. Our findings illustrated that combined use of ELISA and western blotting based on rHc-HCA59 is a powerful tool for early detection of *H. contortus* infection in goats.

**Abstract:**

*Haemonchus contortus* is recognized as one of the important health problems in small ruminants, leading to reduced production and economic loss for farmers worldwide. Prepatent diagnosis of *H. contortus* infection is crucial to improve control strategies as this helminth may remove up to one-fifth of total erythrocytes and may cause anemia, edema, diarrhea, and ultimately death in young animals. In this study, one of the excretory and secretory products, rHc-HCA59, was purified and used as antigen to detect specific antibodies in *H. contortus* infected goats during prepatent stage of infection using indirect enzyme linked immunosorbent assay (ELISA) as screening test. All goats (*n* = 38) were housed indoor, experimentally infected with 8000 infective larvae (L3) of *H. contortus*, and serum samples were collected prior to infection and at 14th day of infection. Immunoblotting was performed to confirm the results of indirect ELISA, evaluate the cross reactivity against rHc-HCA59 in sera of most common co-infecting parasites and rectify the false negative samples. Furthermore, three different batches of rHc-HCA59 were produced to evaluate the repeatability of ELISA. No eggs were detected in feces of all goats collected at 7th and 14th day of infection but, *H. contortus* eggs were detected at 21 days post infection in the feces. Indirect ELISA performed in this study showed 87% sensitivity and 100% specificity. The western blot analysis confirmed immunoreactivity in serum samples which scored positive in indirect ELISA and recognized the samples as negative which had OD_450_ lower than negative cut-off value in indirect ELISA. Furthermore, all false negative sera (*n* = 5) that had OD_450_ value between positive and negative cut-off value in rHc-HCA59 based ELISA were clearly positive in western blot. Moreover, no cross-reactivity was detected in ELISA and western blotting against rHc-HCA59 in positive sera of *Toxoplasma gondii, Fasciola hepatica*, and *Trichinella spiralis.* The results of this study concluded that combined use of indirect ELISA and western blotting with rHc-HCA59 is a potential immunodiagnostic tool for the detection of *H. contortus* infection during prepatent period in goats.

## 1. Introduction

Small ruminant farming industry contributes one of the major means to develop living standards in numerous developing countries and plays an important role in the national economy [1]. Among small ruminant species, goats are particularly vulnerable to gastrointestinal nematodes in humid, tropical, and subtropical regions and are particularly overwhelmed by *Haemonchus contortus* (*H. contortus*) [2]. It is an important blood sucking parasite found in abomasum that may remove about 0.05 mL of blood per day by seepage from lesions [3]. *H. contortus* infection leads to significant economic losses to small ruminant farming industry as a consequence of high mortality and morbidity [1].

The accurate and prepatent diagnosis of *H. contortus* infection is crucial for epidemiological surveillance and control. Prepatent stage detection of *H. contortus* infection has not been attempted in goats, even though both immature worm and fourth larval stage are blood sucking. The current diagnosis of hemonchosis mainly relies on the use of traditional fecal egg counts and serological-based methods, such as FAMACHA (eye-lid coloration) and blood packed cell volume [4]. It is difficult to detect *H. contortus* eggs in feces before the third week of post infection (21–25 days) [5]. *H. contortus* starts blood feeding on 11th day post infection (PI) [4]. Until the infection shows clinical signs, young animals suffer from anemia, diarrhea, edema, weight loss, severe frailty, and ultimately death [6]. These methods are often time-consuming, inaccurate, unreliable, and laborious to perform [7]. 

Immunodiagnosis provides possible avenues to overcome current limitations and develop improved diagnostic assay for detection of *H. contortus*. The immunodiagnosis of *H. contortus* is dependent on direct and indirect techniques. To detect specific antibodies, indirect methods are used. ELISA or western blotting can be used to detect anti-*Haemonchus* antibodies. These techniques are based on target antigens (secreted, purified native, whole parasite extract, or recombinant proteins) being immobilized on a solid support, followed by incubation with serum containing antigen-specific antibodies. The use of recombinant antigens in the design of a specific diagnostic technique facilitates the development of highly sensitive and specific assays that display a high antigen concentration and reduce or eliminate background reactions. Easy production of antigens in expression systems leads to simple and efficient antigen development which can reduce the production costs associated with diagnosis [8].

Excretory and secretory products (ESPs) are produced and released by the parasites during infection [9]. *H. contortus* ESPs (HcESPs) contain many proteins that can perform various functions including host immune response [10]. In previous study, ESPs have been reported as diagnostic antigen for prepatent detection of *H. contortus* infection in sheep [11]. Hepatocellular carcinoma-associated antigen 59 (HCA59) was identified as one of HcESPs and can be isolated from different larval stages of this parasite [12]; however, its diagnosis potency is still unknown. HCA59 belongs to TLS1 (Telomere length and silencing protein 1) family which is potential candidate for immunological applications and provides the molecular features for understanding tumorigenicity [13]. 

The aim of the current study was to evaluate the combined use of ELISA and western blotting to detect specific antibodies against the antigen of *H. contortus* during prepatent stage of infection. Early diagnosis of this infection will assist in treating carrier animals quickly with suitable anthelmintics.

## 2. Material and Methods

### 2.1. Recombinant Protein Purification

The recombinant plasmid expression, rHc-HCA59 was provided by Ministry of Education (MOE) joint international Research Laboratory, Preventive Veterinary Medicine, Nanjing Agricultural University (NAU) (GenBank: CDJ80864.1) and purified by standard protocol described previously [14]. Briefly, transformation of recombinant plasmids into *E.coli* BL21 (DE3) was performed and cultured in ampicillin (100 µg/mL) containing LB medium (Luria Bertini). After that, induction of protein expression was done at 37 °C using IPTG (Isopropyl β-D-thiogalactopyranoside; Sigma-Aldrich) to make OD_600_ 0.6. Culture was centrifuged at 4500 rpm for 15 min and supernatant was discarded. Pallet was lysed by using lysozyme (10 µg/mL Sigma-Aldrich) followed by sonication. 12% (w/v) SDS-PAGE (sodium dodecyl sulfate-polyacrylamide gel electrophoresis) was used to examine the sonication products. Manufacturer’s instructions were practiced to purify recombinant protein using Ni^2+^ nitrilotriacetic acid column (GE Healthcare, Chicago, IL, USA). The rHc-CS protein purity and quantification were performed by SDS-PAGE with Coomassie blue stain and Bradford method respectively [15]. Protein toxins were removed utilizing Toxin Eraser^TM^ Endotoxin Removal kit (GenScript, Piscataway, NJ, USA).

### 2.2. Study Populations

Local crossbred goats aged 4 to 6 months (*n* = 38) were housed indoors and provided with whole shelled corn, hay and water ad libitum. All goats were dewormed with Levamisole (8 mg/kg body weight) to eliminate the naturally attained infection with helminthes at 2-week intervals. Microscopic analysis of fecal samples was performed twice per week for helminth eggs and helminth-free goats were used in further experiments. All goats were experimentally infected orally with 8000 L3 of *H. contortus*. Blood samples were collected from all goats prior to challenging infection (negative) and at 14th post infection day (prepatent stage). Sera were prepared and stored at −20 °C. Serum samples from goats with a positive diagnosis for *Toxoplasma gondii* (*T. gondii*), *Fasciola hepatica* (*F. hepatica*), and *Trichinella spiralis* (*T. spiralis*), provided by MOE joint international Research Laboratory, Preventive Veterinary Medicine NAU, were also used for the evaluation of the ELISA specificity.

Feces samples (*n* = 38) from all goats were collected at 7th and 14th post infection day (prepatent stage) and conventional fecal egg counting was performed with the McMaster method as described previously [16]. McMaster egg counting was based on detection of *H. contortus* eggs in 3 g of feces dispersed in saturated NaCl (42 mL), providing a diagnostic sensitivity of 50 epg (eggs per gram). At the end of the experiment, all goats were confirmed for the presence of *H. contortus* eggs in feces then dewormed to use in another experiment.

### 2.3. Western Blotting Assay

Western blotting plays preliminary role in selection of target protein to diagnose the infection of *H. contortus* and to evaluate the immunogenicity and immunoreactivity of antigen [17]. Western blotting analysis was performed to detect *H. contortus*-specific antibody responses in sera of experimentally infected goats using recombinant *H. contortus* HCA59 (rHc-HCA59). The purified rHc-HCA59 was first deposited in SDS-PAGE wells individually, then transferred onto PVDF (polyvinyl difluoride membrane, Millipore, Billerica, USA) with the help of semi dry system (Novablot Hoefer, USA) and transfer solution (Tris 48 mM, glycine 39 mM, SDS 0.0375%, methanol 20%). The membrane was blocked with 5% skimmed milk diluted in TBS-T (Tris-buffered saline containing 0.05% Tween 20) for 2 h at 37 °C. The PVDF membrane was cut, washed (three times) and incubated with anti-*H. cortortus* 1:100 diluted goat serum (primary antibody) for 2 h at 37 °C. Following three washes with TBS-T the strips were incubated with 1:5,000 diluted secondary antibodies, horseradish peroxidase (HRP) conjugated rabbit anti-goat IgG (Sigma, Hilden, Germany). Subsequently, the strips were washed five times and immunoreactions were observed by virtue of substrate (Tanon™ High-sig ECL Western Blotting, Shanghai, China). Western blotting also helped to check the specificity of rHc-HCA59 against infected sera of *T. gondii*, *F. hepatica*, and *T. spiralis* as primary antibody.

### 2.4. Development of Indirect ELISA

To standardize the indirect ELISA development, different concentrations of rHc-HCA59 (65 to 1.01 μg/mL) and serum dilutions (1:25; 1:50; 1:100; 1:200) were optimized. Negative and positive sera of *H. contortus* were used as controls. The highest P/N (OD_450_ of infected serum/OD_450_ of non-infected serum) value (>2.1) was used to optimize the experimental conditions. Indirect ELISA with optimal conditions of was performed as discussed previously [18]. Briefly, the reaction was performed in 96-well flat bottom without lid high binding plates (Costar, Cambridge, MA, USA). The plate wells were coated with 100 μL of optimum concentration diluted antigen in 0.05 M carbonate buffer solution (CBS; pH 9.6) and incubated overnight at 4 °C. The next day, all wells were blocked with 100 μL BSA (bovine serum albumin; 5%) for 2h at 37 °C. Serum samples diluted 1:50 in BSA were added in triplicate and the plates were again incubated for 2 h at 37 °C. After this duration, diluted secondary antibody in 5% BSA (HRP conjugated rabbit anti-goat IgG; 1:6000) was added and incubated again at 37 °C for 1 h. After each incubation period, plates were washed three times with TBS-T. Finally, plates were washed five times and 100 μL of tetramethylbenzidine (TMB) was added in each well and incubated for 10 min in the dark at room temperature. The color reaction was stopped by adding 100 μL/well of 2M H_2_SO_4_. The optical density (OD) of wells was read at a wavelength of 450 nm using micro plate reader (Thermo Fischer Scientific, Waltham, MA, USA).

### 2.5. Determination of Cut-Off Value

A total 38 serum samples from non-infected goats were evaluated to determine the positive cutoff value from mean OD_450_ of known negative sera + 3 standard deviation [19]. While, negative cut-off value was assessed from mean OD_450_ of known negative sera + 2 standard deviation [20]. The experiment was repeated twice and for the interpretation, any goat sera that had an OD_450_ value greater than the positive cut off value was considered as seropositive while OD_450_ value lower than negative cut-off value was considered as sero-negative [21]. Furthermore, any goat sera which had OD_450_ value between positive and negative cut-off values was considered as false negative/positive.

### 2.6. Calculation of Sensitivity and Specificity

To investigate the further feasibility of indirect ELISA, serum samples (*n* = 38) positive with *H. contortus* were evaluated and diagnostic sensitivity was calculated as “ELISA positive × 100/true *H. contortus*-positive” and serum samples (*n* = 38) from non-infected goats were evaluated to calculate specificity. Additionally, to evaluate the cross-reactivity, 12 serum samples (4 samples for each parasite) positive with *T. gondii*, *F. hepatica*, and *T. spiralis* were used. This experiment was performed in duplicate. To ratify the rHc-HCA59-based indirect ELISA results, all positive, negative, false positive/negative, and other organisms’ sera, were subjected to western blot analyses based on same antigen.

### 2.7. Development of the Western blot-rHc-HCA59

The western blot assay was performed almost as described above. The rHc-HCA59 was deposited in all wells of the SDS-PAGE gel and, after transfer to the nitrocellulose membrane; it was cut to obtain five strips. Each strip was incubated with a 1:100 serum dilution after blocking with 5% skimmed milk for 2 h. The secondary antibody horseradish peroxidase (HRP) conjugated rabbit anti-goat IgG (Sigma, Hilden, Germany) (1:5000) was added to all membranes and incubate for 1 h. Immunoreactions was revealed using substrate (Tanon™ High-sig ECL Western Blotting). All incubation steps were performed at 37 °C and were followed by three washes with TBS-T.

### 2.8. Repeatability of ELISA-rHc-HCA59

In order to evaluate the repeatability of the ELISA, three separate batches of recombinant HCA59 antigen were produced and purified following the methodology described above with distinct purification times, to demonstrate the reproducibility of the recombinant protein expression procedure. Serum samples were selected for testing against each batch of antigen and the averages, and standard deviations, of the O.D. at 450 nm were calculated. Of the samples used for this test, one was strongly reactive positive, three were moderately reactive positive, and four were negative serum samples.

## 3. Results

### 3.1. Purification and Western Blotting

**Figure 1:**
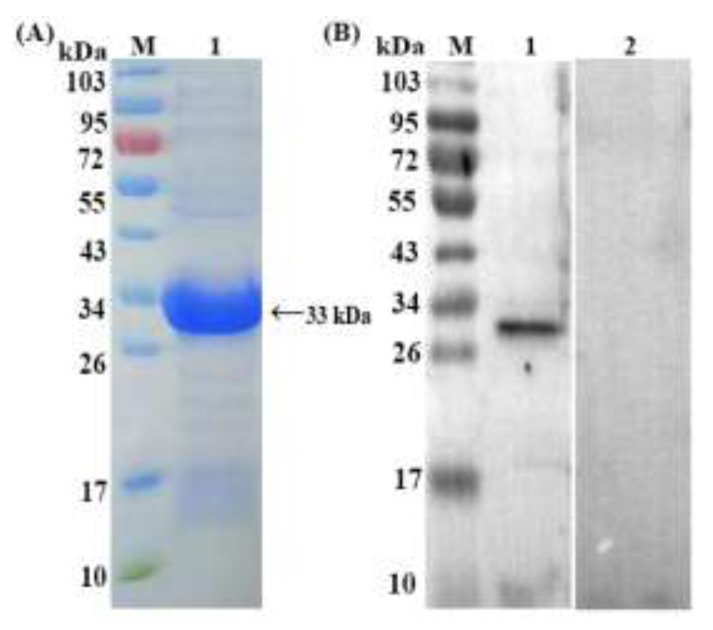
Purification and western blotting of rHc-HCA59 protein. Lane M: standard protein pre stain molecular weight marker. (**A**) Lane 1: Purified rHc-HCA59 protein. (**B**) Lane 1: Antibodies were detected by infected goat sera; Lane 2: membrane incubated with normal goat sera (as control).

**Figure 2:**
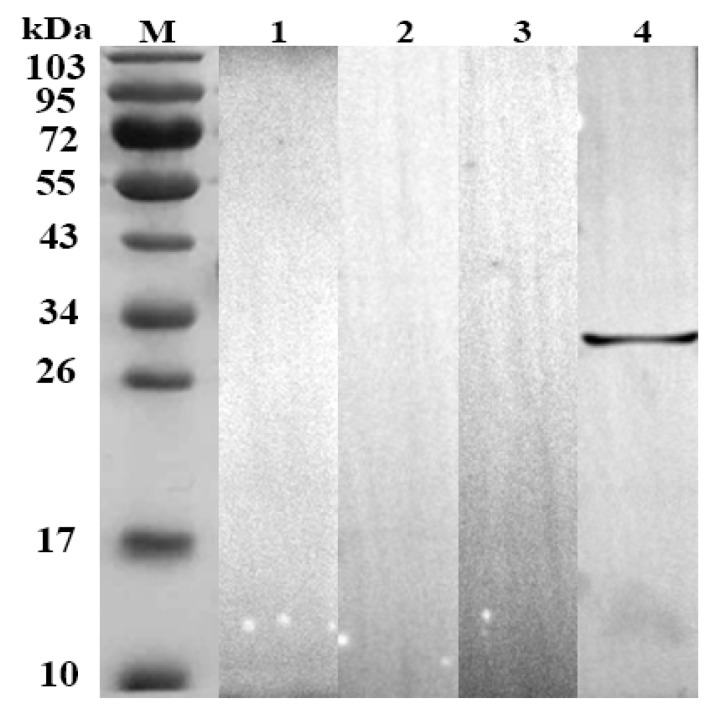
Western blot analysis of rHc-HCA59 antigen to determine cross-reactivity. Lanes shown are as follows: M = standard protein pre stain molecular weight marker; Lane 1: membrane incubated with positive *T. gondii* serum; Lane 2: membrane incubated with positive *F. hepatica* serum; Lane 3: membrane incubated with positive *T. spiralis* serum; Lane 4: rHc-HCA49 was recognized by goat anti-*H. contortus* sera (positive control).

The SDS-PAGE results demonstrated the presence of distinct bands, rHc-HCA59 showed a molecular mass of 33 kDa (Figure 1A). The same band was observed in the Western blotting and confirmed the presence of band that corresponded to the protein of interest (Figure 1B). Previous study has described the cloning of the 426 bp corresponding to the rHc-HCA59 protein gene from *H. contortus* and reported that the recombinant protein presented as a distinct molecular mass, 33 kDa [14]. This corroborates with the results observed through SDS-PAGE and western blot in the present study (Figure 1). Furthermore, the rHc-HCA59 antigen did not show any cross-reaction with goat anti-sera of *T. gondii*, *F. hepatica*, and *T. spiralis* (Figure 2).

### 3.2. Microscopic Examination

McMaster fecal egg counts (FECs) could not detect the eggs of *H. contortus* in fecal samples of all infected goats (*n* = 38) collected at 7th and 14th day of infection. While, *H. contortus* eggs were detected at the 21st day of post infection in feces.

### 3.3. Cut-Off Value of Indirect ELISA

The positive cut-off value of indirect ELISA was 0.371 as determined by mean value (0.256 ± 0.004) plus three multiplied by standard deviations (3 × 0.038) of the OD_450_ values obtained from 38 known negative goat sera. While, the negative cut-off value of indirect ELISA was 0.332 as determined by mean value (0.256 ± 0.004) plus two multiplied by standard deviations (2 × 0.038) of the OD_450_ values.

### 3.4. Development and Serodiagnostic Potential of Indirect ELISA

**Figure 3:**
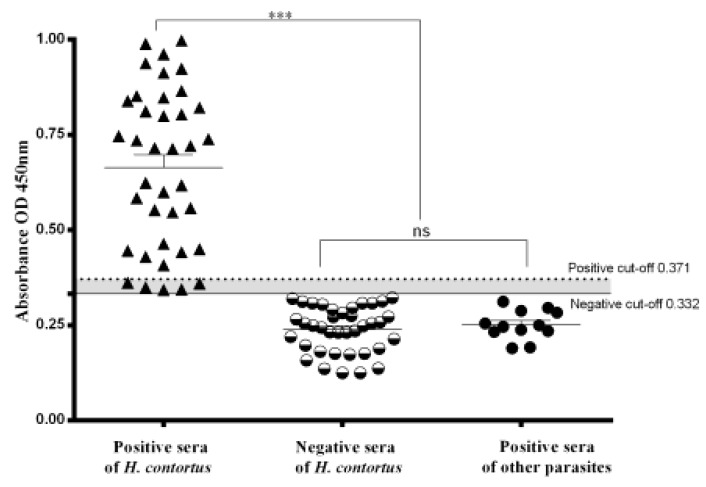
Sensitivity, specificity, and cross-reactivity of indirect-ELISA based on rHc-HCA59. The dotted horizontal line represents the positive cut-off value (OD_450_ = 0.371). The solid horizontal line represents the negative cut-off value (OD_450_ = 0.332). Statistically significant differences were observed between *H. contortus*-positive sera and the other organisms’ sera (*T. gondii*, *F. hepatica*, and *T. spiralis*-positive) and *H. contortus*-negative sera. No significant difference was noted between the *H. contortus*-negative and other parasites-positive serum samples.

The optimum concentration of rHc-HCA59 antigen and optimum dilution of serum were 0.81 μg/well and 1:50, respectively (Table 1). The rHc-HCA59 has definite potential for serological diagnosis through the established indirect ELISA. The indirect ELISA showed 87% sensitivity against well-characterized positive sera and 100% specificity against helminth free sera. Total 88 serum samples (38 positive for *H. contortus* collected at prepatent stage of infection; 38 negative; 12 positive for other co-infection parasites) were used to evaluate the diagnostic potential of indirect ELISA. Of which, OD_450_ of 28 sera samples were observed above positive cut-off value and scored positive (OD_450_ > 0.371). OD_450_ value of 38 negative sera samples was observed below the negative cut-off line and scored negative (OD_450_ < 0.332) in ELISA-rHc-HCA59. Moreover, five sera samples collected at the prepatent stage from *H. contortus* positive goats, which were negative in microscopic examination, showed OD_450_ value between the positive and negative cut-off line and considered as false negative. While all *T. gondii*, *F. hepatica*, and *T. spiralis-*positive sera tested negative (OD_450_ < 0.332) in the developed rHc-HCA59 ELISA, providing evidence of test specificity. Significant differences (*p* < 0.05) in mean OD values between *H. contortus* infected goats’ sera (0.681 ± 0.012) and uninfected sera (0.268 ± 0.004) were observed by t-test. While, no significant difference between negative and other parasites’ serum samples were observed (Figure 3). The results indicated that five serum samples showed false negative OD_450_.

### 3.5. Western Blotting for Ratification of Indirect ELISA Results

**Figure 4:**
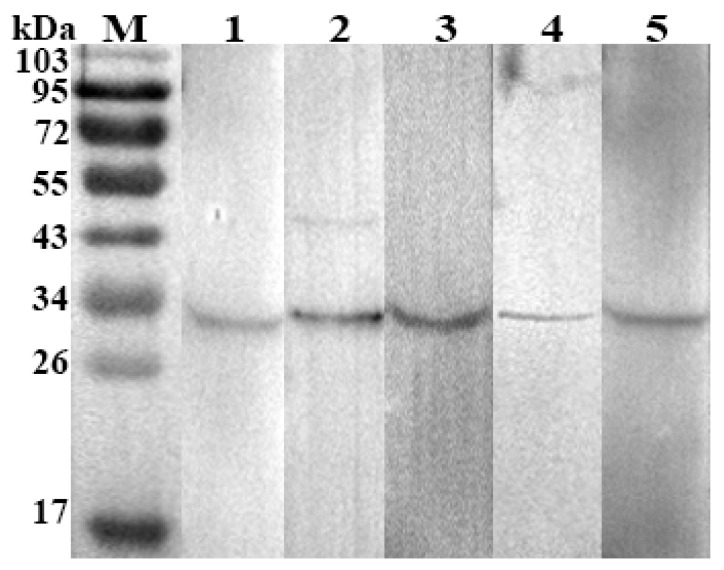
Western blot analyses of the goat serum samples that scored false negative in the ELISA-rHc-HCA59. M: standard protein pre stain molecular weight marker; Lanes 1–5: Serum samples with discrepant ELISA results. Specific antibodies against rHc-HCA59 were detected in all goats’ sera.

To ratify the indirect ELISA results, all positive (*n* = 33), negative (*n* = 38), false negative (*n* = 5) and other organisms’ sera (*n* = 12), were subjected to rHc-HCA59-based western blot. The results of western blot analysis confirmed those samples as positive having OD_450_ value greater than positive cut-off value and recognised the samples as negative which had OD_450_ lower than negative cut-off value in indirect ELISA. Furthermore, all false negative sera that had OD_450_ value between positive and negative cut-off value in rHc-HCA59 based ELISA were clearly positive in western blot (Figure 4). While no cross-reactivity was observed in sera positive for *T. gondii*, *F. hepatica*, and *T. spiralis.*

### 3.6. Test Repeatability

**Figure 5:**
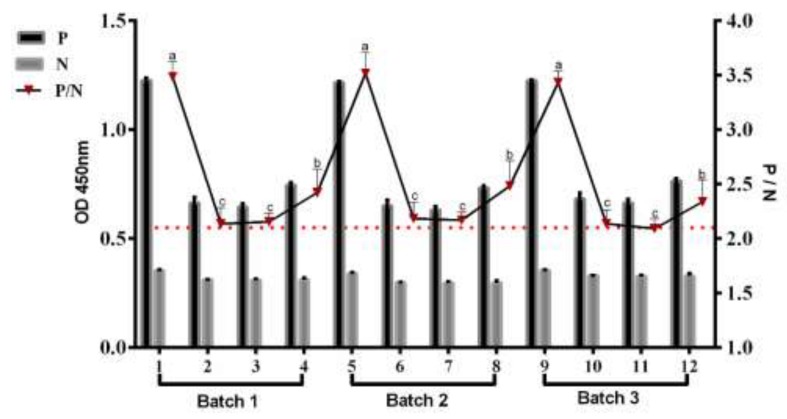
Evaluation of different batches of rHc-HCA59 protein production used as antigen in the indirect ELISA development and tested with positive and negative sera for H. contortus. The P/N (OD_450_ value of positive serum/OD_450_ value of negative serum) value of each batch was shown on each bar (right Y axis), P/N value (>2.1) was determined to be positive results. Sample 1, 5, and 9: strongly reactive positive and showed higher P/N value, the remaining samples were moderately positive. No significant differences were observed between the same samples of different batches.

Three different protein batches were used as coating antigens for testing the rHc-HCA59-ELISA and the results from all three were similar (*p* > 0.05) and thus, indicate antigen stability. The optical density (OD) values derived from the checkerboard were calculated and showed as the positive to negative (P/N) ratio [22]. The high P/N (OD_450_ of infected serum/OD_450_ of non-infected serum) value (>2.1) was observed (Figure 5).

## 4. Discussion

*H. contortus* is prevalent wherever small ruminants are raised, but the greatest economic losses occur in warm and tropical areas. Detection of *H. contortus* infection in small ruminants during the 15-day prepatent period is essential for effective control of this disease on farms and field [23]. The current diagnosis of haemonchosis mainly relies on the use of traditional fecal egg counts [4]. After ingestion of infective larva (L3) by host, HCI and pepsin triggered exsheathment for L3 migration to abomasum where L3 develops into L4 stage, which feeds on blood followed by final adult development. It takes approximately 3 weeks post-infection. It is not possible to detect *H. contortus* eggs in feces before week 3 post-infection (21–25 days) in small ruminants [24]. That is why in this study, no eggs of *H. contortus* were detected in fecal samples of all infected goats collected at the 7th and 14th days of infection.

Previous study that focused on the development of potential diagnostic assays was not limited to *H. contortus* detection, but also focused on development of vaccine against haemonchosis and interaction between parasite molecules and host cells [25,26,27]. Different antigens—such as somatic antigens, larval antigen, and crude antigen—were evaluated for immunodiagnosis against *H. contortus* infection [5,28,29]. However the cross reactivity among different helminthes and low sensitivity are the limiting factors of these antigens. 

Recently, modulatory effects of rHc-HCA59 on PBMCs (peripheral blood mononuclear cells ) and maturation of monocyte-derived dendritic cells were evaluated in goat and reported that rHc-HCA59 could be recognized by *H. contortus* infected goat sera [14]. These features make this antigen an interesting candidate for use in diagnostic assays, such as ELISA and western blotting. In this study, rHc-HCA59 was used as diagnostic antigen for development of indirect ELISA as well as for detection of specific antibody during prepatent period. Previously, different ELISA based methods have been reported to detect anti-*H. contortus* antibodies in naturally and/or experimentally infected sheep sera [23,30,31]. The ELISA-rHc-HCA59 developed in this study showed 87% sensitivity and 100% specificity results and thus proves that rHc-HCA59 can be used as diagnostic antigen to powerfully detect specific antibodies during prepatent period of *H. contortus* infection in goat. In contrast to ELISA of this study, DAS-ELISA (double antibody sandwich ELISA) and dot-ELISA were developed to detect *Babesia gibsoni* [21] using truncated recombinant BgSA1, achieved sensitivity of 100% and 91.9% respectively, and specificity of 97.4% and 84.2% respectively. Secretion time of antigen may influence antigenicity, specificity and sensitivity during prepatent stage of infection. It has been reported that rHc-HCA59 releases at 7th and 14th day of infection [12]. Therefore, this feature gives this antigen potential to detect specific antibody during prepatent stage of infection. Furthermore, rHc-HCA59 did not show any cross reactivity against co-infecting parasites, that is why this antigen showed high specificity. In contrast to this study, previous study showed cross-reactivity against *H. contortus* infected sheep sera using recombinant protein recIgE1-2 [32].

In the present study, western blot assay was used to complement the results achieved through ELISA and to support the diagnosis of *H. contortus* infection further. The combination of indirect ELISA and western blotting can be an effective tool to increase sensitivity of serodiagnosis of infection [33]. In this study, rHc-HCA59-based western blotting was used to confirm the results of screening indirect ELISA assay and results confirmed the immunoreactivity in all positive sera that had higher OD value than the positive cut-off value (OD_450_ > 0.371). While, this confirmatory test did not show any cross-reactivity against rHc-HCA59 in positive sera of other organisms and negative sera (control) during the ratifying process of indirect ELISA.

Furthermore, five samples with OD_450_ between positive and negative cut off values were negative in ELISA but in western blotting, specific antibodies were detected in these samples. One possible reason for this incongruity may be the blocking buffer used in the assay. The results of antibody assay significantly depend on the buffer system used [34]. The blocking buffer used in indirect ELISA was 5% BSA while, western blot analysis used 5% skimmed milk. Another possible cause for this discordance may be the concentration of antibody used in both assays. Lastly, false negative reactions possibly occurred due to the lower dilutions of secondary antibody selected in attempt to make lower OD values of normal goats. The concentration of secondary antibody used in indirect ELISA was 1:6000, while 1:5000 dilutions were used in western blot assay. False negative results are really undesirable, especially if a lot of animals are misdiagnosed. In this study, rHc-HCA59-based western blot assay provided effective support to eliminate the level of false negatives. Hence, the indirect ELISA applied together with the western blotting assay may avoid or decrease false negative results. Further study for the investigation of *H. contortus* prevalence is needed on a large scale for application of this assay in the field. The rHc-HCA59 produced in current study was expressed in the soluble fraction and was recoverable from culture easily. Different productions of rHc-HCA59 showed almost same yield and the same ELISA test results and proved the stability of antigen.

## 5. Conclusions

The indirect ELISA developed here with rHc-HCA59 as an antigen which can detect anti *H. contortus* antibodies in goat serum at good sensitivity and specificity. The western blot assay developed with rHc-HCA59 was able to detect antibodies that were false negatives in the ELISA test and showed increased sensitivity. The results of this study concluded that combined use of indirect ELISA and western blotting with rHc-HCA59 is a potential immunodiagnostic tool to detect *H. contortus* infection during the prepatent period in goats.

## Figures and Tables

**Table 1:** Determination of the optimal rHc-HCA59 coating concentration and serum dilution for indirect-ELISA.

Antigen Dilution	OD_450_	Antibody Dilution			
μg/well		1:25	1:50	1:100	1:200
6.5	(P)	1.631	1.517	0.738	0.528
	(N)	1.428	1.204	0.541	0.273
	P/N	1.142	1.26	1.364	1.934
3.25	(P)	1.467	1.291	0.679	0.623
	(N)	0.673	0.584	0.379	0.321
	P/N	2.180	2.211	1.792	1.941
1.62	(P)	1.248	1.074	0.685	0.595
	(N)	0.536	0.456	0.304	0.251
	P/N	2.328	2.355	2.253	2.371
0.81	(P)	1.256	1.102	0.613	0.465
	(N)	0.393	0.269	0.211	0.201
	P/N	3.196	**4.097**	2.905	2.313
0.40	(P)	1.022	0.853	0.538	0.407
	(N)	0.359	0.235	0.199	0.187
	P/N	2.847	3.63	2.704	2.176
0.2	(P)	0.919	0.738	0.427	0.371
	(N)	0.335	0.221	0.194	0.178
	P/N	2.743	3.339	2.201	2.084
0.1	(P)	0.611	0.432	0.338	0.301
	(N)	0.312	0.204	0.196	0.188
	P/N	1.958	2.118	1.724	1.601

P: OD450 Value of positive serum; N: OD450 value of negative serum; Bold represents the optimum conditions for this indirect-ELISA method, the highest P/N value is 4.097.

## Abbreviations

rHc-HCA59	Recombinant Hepatocellular Carcinoma-associated antigen 59
PI	Post infection
HcESPs	Excretory and secretory products of *Haemonchus contortus*
NAU	Nanjing Agricultural University
LB	Luria Bertini
AEC	Animal Ethics Committee
IPTG	Isopropyl-b-D-thiogalactopyranoside
SDS-PAGE	Sodium dodecyl sulfate-polyacrylamide gel electrophoresis
PVDF	Polyvinyl difluoride membrane
HRP	Horseradish peroxidase
TMB	Tetramethylbenzidine
OD	Optical density
P/N	Positive to negative
BSA	Bovine serum albumin
TBS-T	Tris-Buffered Saline containing 0.05% Tween 20
MW	Molecular weight
kDa	Kilodalton
PBMCs	Peripheral blood mononuclear cells

## References

[1] Alam M.B.B., Omar A.I., Faruque M.O., Notter D.R., Periasamy K., Mondal M.M.H., Sarder M.J.U., Shamsuddin M., Cao J., Du X. (2019). Single nucleotide polymorphisms in candidate genes are significantly associated with resistance to Haemonchus contortus infection in goats. J. Anim. Sci. Biotechnol..

[2] Traoré A., Notter D.R., Soudre A., Kaboré A., Álvarez I., Fernández I., Sanou M., Shamshuddin M., Periasamy K., Tamboura H.H. (2017). Resistance to gastrointestinal parasite infection in Djallonké sheep. Animal.

[3] Qamar M.F., Maqbool A., Khan M.S., Ahmad N., Muneer M.A. (2009). Epidemiology of haemonchosis in sheep and goats under different managemental conditions. Vet. World.

[4] Rodríguez A.V., Goldberg V., Viotti H., Ciappesoni G. (2015). Early detection of *Haemonchus contortus* infection in sheep using three different faecal occult blood tests. Open Vet. J..

[5] Kandil O.M., Eid N.A., Elakabawy L.M., Abdelrahman K.A., Helal M.A. (2015). Immunodiagnostic Potency of Different *Haemonchus contortus* Antigens for Diagnosis of Experimentally and Naturally Haemonchosis in Egyptian Sheep. APG.

[6] Squires J.M., Ferreira J.F.S., Lindsay D.S., Zajac A.M. (2011). Effects of artemisinin and Artemisia extracts on Haemonchus contortus in gerbils (*Meriones unguiculatus*). Vet. Parasitol..

[7] Gasser R.B., Bott N.J., Chilton N.B., Hunt P., Beveridge I. (2008). Toward practical, DNA-based diagnostic methods for parasitic nematodes of livestock - Bionomic and biotechnological implications. Biotechnol. Adv..

[8] Lugovskaya N.N., Scherbakov A.V., Yakovleva A.S., Tsyvanyuk M.A., Mudrak N.S., Drygin V.V., Borisov A.V. (2006). Detection of antibodies to avian infectious bronchitis virus by a recombinant nucleocapsid protein-based enzyme-linked immunosorbent assay. J. Virol. Methods.

[9] Yatsuda A.P., Krijgsveld J., Cornelissen A.W.C.A., Heck A.J.R., De Vries E. (2003). Comprehensive analysis of the secreted proteins of the parasite *Haemonchus contortus* reveals extensive sequence variation and differential immune recognition. J. Biol. Chem..

[10] Gadahi J.A., Yongqian B., Ehsan M., Zhang Z.C., Wang S., Yan R.F., Song X.K., Xu L.X., Li X.R. (2016). *Haemonchus contortus* excretory and secretory proteins (HcESPs) suppress functions of goat PBMCs *in vitro*. Oncotarget.

[11] Schallig H.D.F.H., Leeuwen M.A.W.V., Hendrikx W.M.L. (1994). Immune responses of Texel sheep to excretory/secretory products of adult *Haemonchus contortus*. Parasitology.

[12] Gadahi J.A., Wang S., Bo G., Ehsan M., Yan R.F., Song X.K., Xu L.X., Li X.R. (2016). Proteomic Analysis of the Excretory and Secretory Proteins of *Haemonchus contortus* (HcESP) Binding to Goat PBMCs In Vivo Revealed Stage-Specific Binding Profiles. PLoS ONE.

[13] Wang Y., Han K.-J., Pang X.-W., Vaughan H.A., Qu W., Dong X.Y., Peng J.R., Zhao H.T., Rui J.A., Leng X.S. (2002). Large scale identification of human hepatocellular carcinoma-associated antigens by autoantibodies. J. Immunol..

[14] Wang Q.Q., Wu L.Y., Hasan M.W., Lu M.M., Wang W.J., Yan R.F., Xu L.X., Song X.K., Li X.R. (2019). Hepatocellular carcinoma-associated antigen 59 of *Haemonchus contortus* modulates the functions of PBMCs and the differentiation and maturation of monocyte-derived dendritic cells of goats in vitro. Parasites Vectors.

[15] Han K., Xu L., Yan R., Song X., Li X. (2011). Cloning, expression and characterization of NAD^+^-dependent glyceraldehyde-3-phosphate dehydrogenase of adult *Haemonchus contortus*. J. Helminthol..

[16] Ljungström S., Melville L., Skuce P.J., Höglund J. (2018). Comparison of Four Diagnostic Methods for Detection and Relative Quantification of *Haemonchus contortus* Eggs in Feces Samples. Front. Vet. Sci..

[17] El-Hassan E.M., El-Bahr S.M. (2012). Antigenic and immunogenic components of *Haemonchus longistipes* identified by western Immunobloting. Am. J. Biochem. Biotechnol..

[18] Tankaew P., Srisawat W., Singhla T., Tragoolpua K., Kataoka Y., Sawada T., Sthitmatee N. (2018). Comparison of two indirect ELISA coating antigens for the detection of dairy cow antibodies against *Pasteurella multocida*. J. Microbiol. Methods.

[19] Anuracpreeda P., Chawengkirtikul R., Tinikul Y., Poljaroen J., Chotwiwatthanakun C., Sobhon P. (2013). Diagnosis of *Fasciola gigantica* infection using a monoclonal antibody-based sandwich ELISA for detection of circulating cathepsin B3 protease. Acta Trop..

[20] Akao T., Kakehi Y., Wu X., Kinoshita H., Takahashi T., Ogawa O., Kato T., Yoshida O. (1997). Semi-Quantitative Analysis of Telomerase Activity of Exfoliated Cells in Urine of Patients with Urothelial Cancers. Urol. Oncol..

[21] Mandal M., Banerjee P.S., Kumar S., Garg R., Ram H., Kundu K., Raina O.K. (2014). Development and evaluation of serodiagnostic assays with recombinant BgSA1 of *Babesia gibsoni*. Vet. Parasitol..

[22] Tankaew P., Singh-La T., Titaram C., Punyapornwittaya V., Vongchan P., Sawada T., Sthitmatee N. (2017). Evaluation of an In-house indirect ELISA for detection of antibody against haemorrhagic septicemia in Asian elephants. J. Microbiol. Methods.

[23] Prasad A., Nasir A., Singh N. (2008). Detection of anti-Haemonchus contortus antibodies in sheep by dot- ELISA with immunoaffinity purified fraction of ES antigen during prepatency. Indian J. Exp. Biol..

[24] Molina J.M., Martín S., Hernández Y.I., González J.F., Ferrer O., Ruiz A. (2012). Immunoprotective effect of cysteine proteinase fractions from two *Haemonchus contortus* strains adapted to sheep and goats. Vet. Parasitol..

[25] Han K., Xu L., Yan R., Song X., Li X. (2012). Vaccination of goats with glyceraldehyde-3-phosphate dehydrogenase DNA vaccine induced partial protection against *Haemonchus contortus*. Vet. Immunol. Immunopathol..

[26] Han K., Xu L., Yan R., Song X., Li X. (2012). Molecular cloning, expression and characterization of enolase from adult *Haemonchus contortus*. Res. Vet. Sci..

[27] Gadahi J.A., Li B., Ehsan M., Wang S., Zhang Z., Wang Y., Hasan M.W., Yan R., Song X., Xu L. (2016). Recombinant *Haemonchus contortus* 24 kDa excretory/secretory protein (rHcES-24) modulate the immune functions of goat PBMCs *in vitro*. Oncotarget.

[28] Gowda A.K.J. (2014). Sero-prevalence of *Haemonchus contortus* infection in sheep by Indirect-ELISA using somatic antigen. J. Parasit. Dis..

[29] Mohmad A., Chandra D., Saravanan B.C., Manjunathchar H.V., Vinodh K.O.R., Fular A., Chigure G., Kaur N., Ghosh S. (2018). Development of a recombinant TaSP-based Dot-ELISA for detection of *Theileria annulata* infection in cattle. Ticks Tick. Borne. Dis..

[30] Li X., Du A., Cai W., Hou Y., Pang L., Gao X. (2007). Evaluation of a recombinant excretory secretory *Haemonchus contortus* protein for use in a diagnostic enzyme-linked immunosorbent assay. Exp. Parasitol..

[31] Lone B.A., Chishti M.Z., Ahmad F., Tak H., Hassan J. (2012). Immunodiagnosis of *Haemonchus contortus* infection in sheep by indirect enzyme linked immunosorbent assay. Ir J. Vet. Res..

[32] Kooyman F.N.J., Van Kooten P.J.S., Huntley J.F., MacKellar A., Cornelissen A.W.C.A., Schallig H.D.F.H. (1997). Production of a monoclonal antibody specific for ovine immunoglobulin E and its application to monitor serum IgE responses to *Haemonchus contortus* infection. Parasitology.

[33] Beck S.T., Leite O.M., Arruda R.S., Ferreira A.W. (2005). Combined use of Western blot/ELISA to improve the serological diagnosis of human tuberculosis. Brazilian J. Infect. Dis..

[34] Waritani T., Chang J., McKinney B., Terato K. (2017). An ELISA protocol to improve the accuracy and reliability of serological antibody assays. MethodsX.

